# Supplementary material for: Visualization of accessible cholesterol using a GRAM domain-based biosensor
Source: Nat Commun. 2023 Oct 25;14:6773. doi: 10.1038/s41467-023-42498-7 (PMC10600248; doi:10.1038/s41467-023-42498-7)
Supplement: Supplementary file 1 — Supplementary Information [file 41467_2023_42498_MOESM1_ESM.pdf]

Supplementary Information

Visualization of accessible cholesterol using a GRAM domain-based biosensor

Dylan Hong Zheng Koh[1], Tomoki Naito[1], Minyoung Na[1], Yee Jie Yeap[1], Pritisha Rozario[1], Franklin L. Zhong[1,2], Kah-Leong Lim[1,3], Yasunori Saheki[1,4]*

[1]Lee Kong Chian School of Medicine, Nanyang Technological University, 308232, Singapore

[2]Skin Research Institute of Singapore (SRIS), Singapore, 308232, Singapore

[3]National Neuroscience Institute, Singapore, 308433, Singapore

[4]Institute of Resource Development and Analysis, Kumamoto University, Kumamoto, 860-0811, Japan

*Address correspondence to: yasunori.saheki@ntu.edu.sg (Y.S.)

- **Supplementary Figures 1–7**
- **Supplementary Table 1**

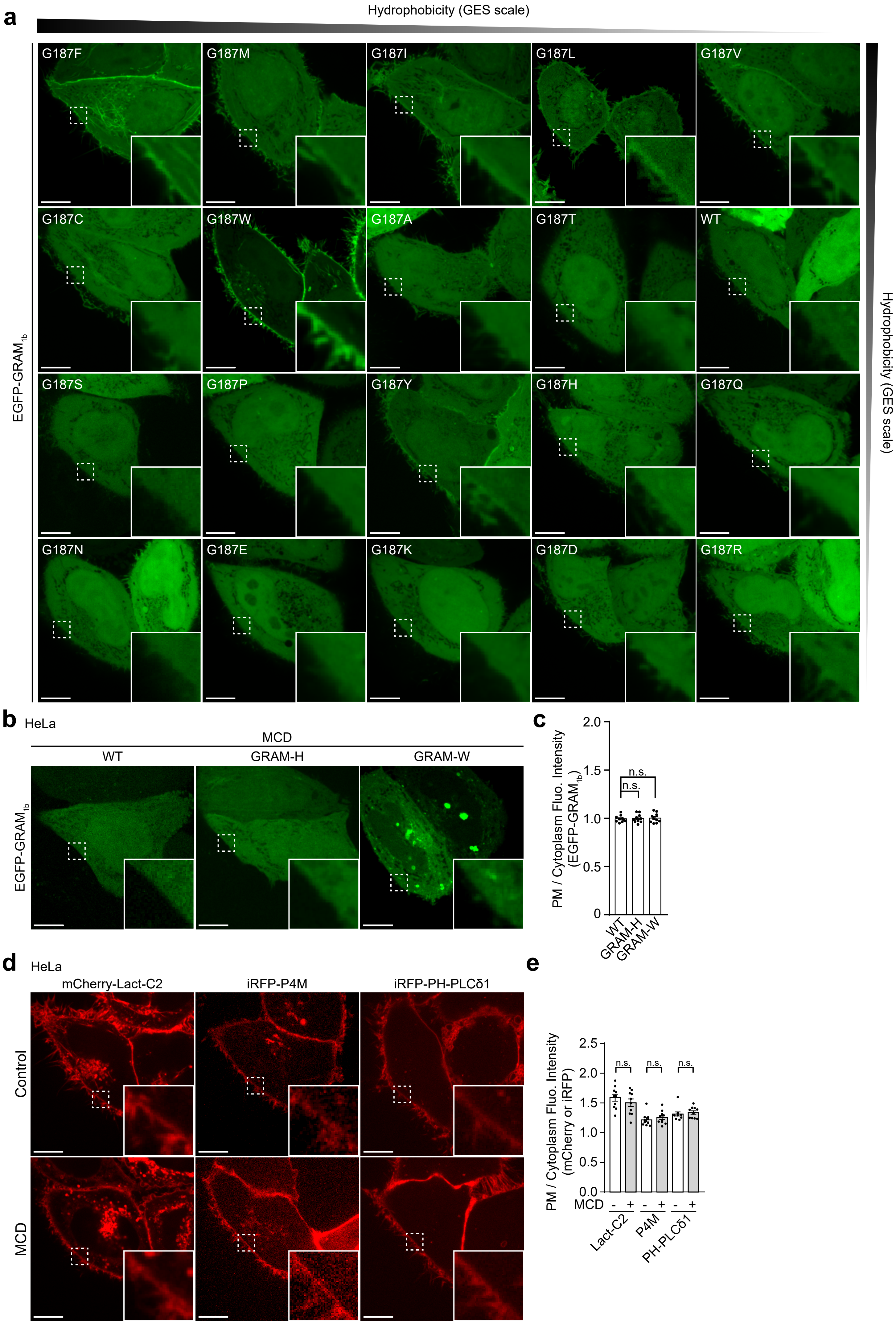
a
Hydrophobicity (GES scale)
EGFP-GRAM1b
G187F
G187M
G187I
G187L
G187V
G187C
G187W
G187A
G187T
WT
G187S
G187P
G187Y
G187H
G187Q
G187N
G187E
G187K
G187D
G187R
Hydrophobicity (GES scale)
b HeLa
MCD
WT
GRAM-H
GRAM-W
EGFP-GRAM1b
c
PM / Cytoplasm Fluo. Intensity (EGFP-GRAM1b)
2.0
1.5
1.0
0.5
0
n.s.
n.s.
WT
GRAM-H
GRAM-W
d HeLa
mCherry-Lact-C2
iRFP-P4M
iRFP-PH-PLCδ1
Control
MCD
e
PM / Cytoplasm Fluo. Intensity (mCherry or iRFP)
2.5
2.0
1.5
1.0
0.5
0.0
n.s.
n.s.
n.s.
MCD
-
+
-
+
-
+
Lact-C2
P4M
PH-PLCδ1

Supplementary Fig. 1. Visualization of accessible cholesterol by a GRAM-W biosensor. a. Confocal images of live HeLa cells expressing mutant versions of EGFP-tagged $GRAM_{1b}$ (EGFP-$GRAM_{1b}$) as indicated. Insets show at higher magnification the regions indicated by white dashed boxes. Quantification of the ratio of PM signals to cytosolic signals of EGFP-$GRAM_{1b}$ is shown in Fig. 1b. Scale bars, 10 µm. **b.** Confocal images of live HeLa cells expressing either wild-type EGFP-$GRAM_{1b}$ (WT), EGFP-tagged GRAM-H (GRAM-H), or EGFP-tagged GRAM-W (GRAM-W) as indicated. Cells were treated with MCD (5 mM) for 20 min before imaging. Insets show at higher magnification the regions indicated by white dashed boxes. Scale bars, 10 µm. **c.** Quantification of the ratio of PM signals to cytosolic signals of EGFP-$GRAM_{1b}$, as assessed by confocal microscopy and line scan analysis from HeLa cells expressing indicated constructs as shown in (b) (mean ± SEM, n = 10 cells for each condition; data are pooled from one experiment; Dunnett's multiple comparisons test, n.s. denotes not significant). **d.** Confocal images of live HeLa cells expressing either mCherry-tagged Lact-C2 (mCherry-Lact-C2), iRFP-tagged P4M (iRFP-P4M), or iRFP-tagged PH-PLCδ1 (iRFP-PH-PLCδ1) as indicated. Cells were treated with or without MCD (5 mM) for 20 min before imaging. Insets show at higher magnification the regions indicated by white dashed boxes. Scale bars, 10 µm. **e.** Quantification of the ratio of PM signals to cytosolic signals of mCherry-Lact-C2, iRFP-P4M, or iRFP-PH-PLCδ1, as assessed by confocal microscopy and line scan analysis from HeLa cells expressing indicated constructs, with or without MCD treatment as shown in (d) (mean ± SEM, n = 10 cells for each condition; data are pooled from one independent experiment; two-tailed unpaired Student's t-test, n.s. denotes not significant).

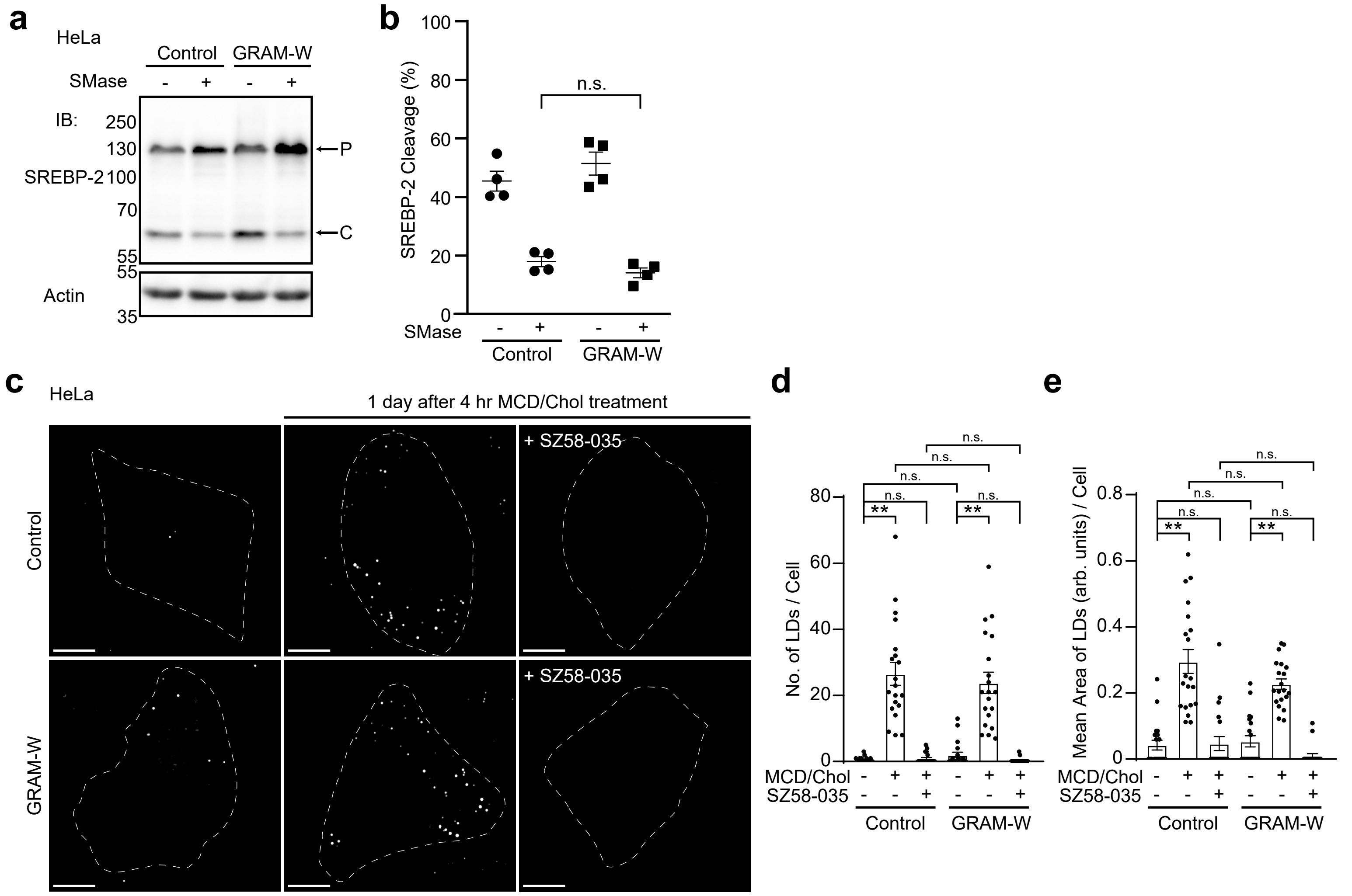

Supplementary Fig. 2. Expression of GRAM-W does not impair intracellular movement of accessible cholesterol. a. Lysates of wild-type HeLa cells (Control) or HeLa cells stably expressing EGFP-GRAM-W (GRAM-W) that were treated with or without 100 mU/ml sphingomyelinase (SMase) for 3 hrs and processed by SDS-PAGE and immunoblotted (IB) with anti-SREBP-2 and anti-actin antibodies. P, precursor form; C, cleaved form. **b.** Quantification of the percentage of the cleaved form of SREBP-2 [cleaved form / (cleave form + precursor form)] as analyzed in (a) (n = 4 for each condition; two-tailed unpaired Student's t-test, n.s. denotes not significant). **c.** Confocal images of fixed wild-type HeLa cells (Control) and HeLa cells stably expressing EGFP-GRAM-W (GRAM-W) that were stained with LipidTOX to visualize lipid droplets (LDs). Cells were treated with MCD/Chol (200 μM) for 4 hours. Medium were then replaced with culture medium with or without ACAT inhibitor, SZ58-035 (10 μM), for 1 day before imaging. White dotted lines are drawn to depict the PM. Scale bars, 10 μm. **d.** Quantification of the number of LDs stained by LipidTOX, as shown in (c) (mean ± SEM, n = 20 cells for each condition; data are pooled from one experiment; Tukey's multiple comparisons test, **P < 0.0001, n.s. denotes not significant). **e.** Quantification of the size of LDs stained by LipidTOX, as shown in (c) (mean ± SEM, n = 20 cells for each condition; data are pooled from one experiment; Tukey's multiple comparisons test, **P < 0.0001, n.s. denotes not significant).

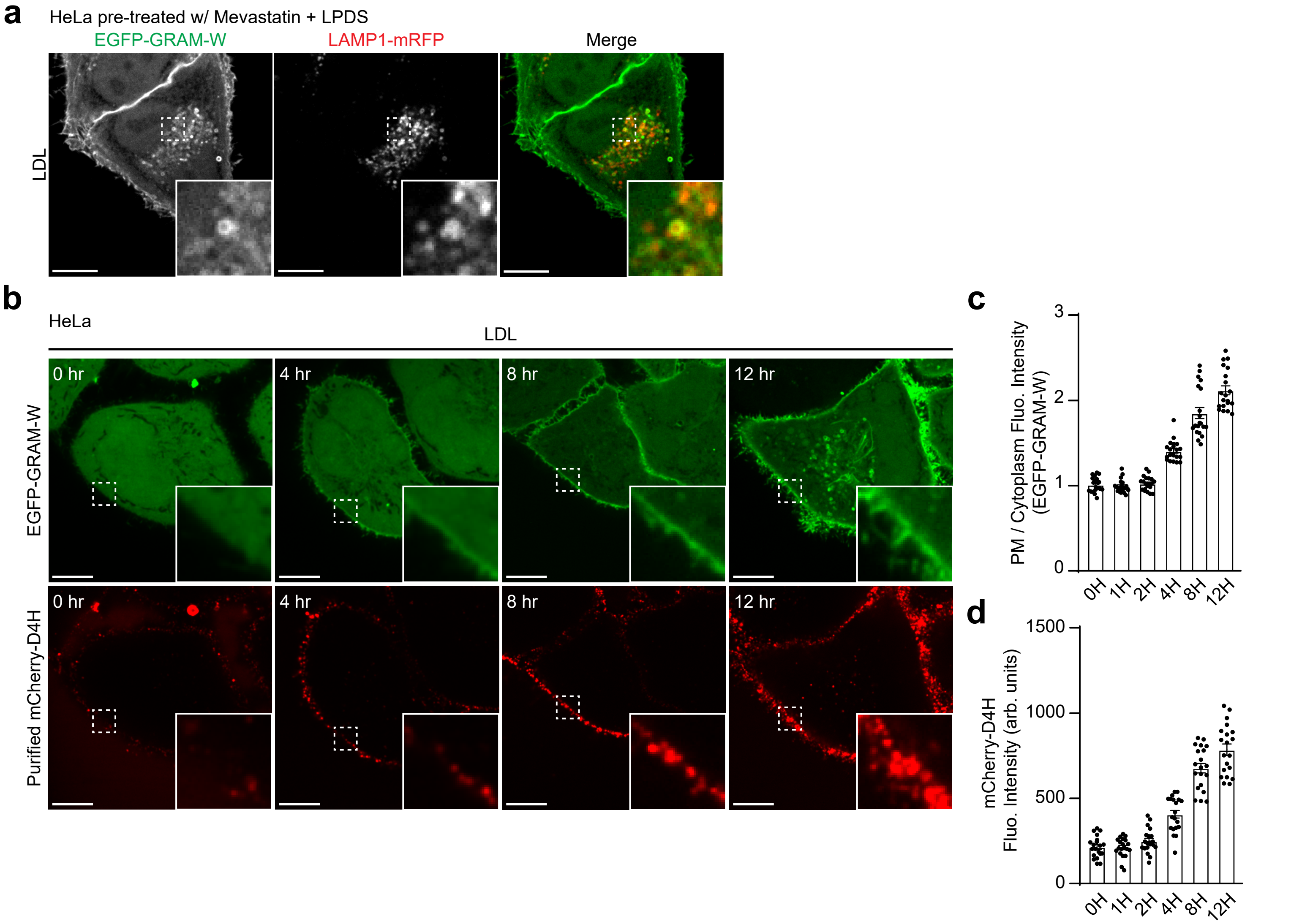

Supplementary Fig. 3. GRAM-W detects LDL-derived accessible cholesterol in lysosomal membranes and the PM. a. Confocal images of live HeLa cells expressing EGFP-GRAM-W and LAMP1-mRFP (lysosomal marker) that were stimulated by low-density lipoprotein (LDL) (50 µg/ml) for 12 hrs. Cells were pre-incubated in medium supplemented with mevastatin (50 µM) and 10% LPDS for 16 hrs to deplete cholesterol. Insets show at higher magnification the regions indicated by white dashed boxes. Same cells as shown in Fig. 2b. Experiments were repeated two times with similar results. Scale bars, 10 µm. **b.** Confocal images of live HeLa cells stably expressing EGFP-GRAM-W that were stimulated by low-density lipoprotein (LDL) (50 µg/ml) for different periods of time as indicated. Cells were pre-incubated in medium supplemented with mevastatin (50 µM) and 10% LPDS for 16 hrs to deplete cholesterol. Live cells were stained with purified mCherry-D4H proteins (15 µg/ml for 10 min) before imaging. Insets show at higher magnification the regions indicated by white dashed boxes. Note the similar timing of the increase in the binding of EGFP-GRAM-W and mCherry-D4H proteins to the PM upon LDL treatment. Scale bars, 10 µm. **c.** Quantification of the ratio of PM signals to cytosolic signals of EGFP-GRAM-W, as assessed by confocal microscopy and line scan analysis as shown in (b) (mean ± SEM, n = 20 cells for each condition; data are pooled from two experiments; Dunnett's multiple comparisons test, $^{**}P < 0.0001$). **d.** Quantification of the mCherry-D4H signals, as assessed by confocal microscopy and line scan analysis as shown in (b) (mean ± SEM, n = 20 cells for each condition; data are pooled from two experiments; Dunnett's multiple comparisons test, $^{**}P < 0.0001$).

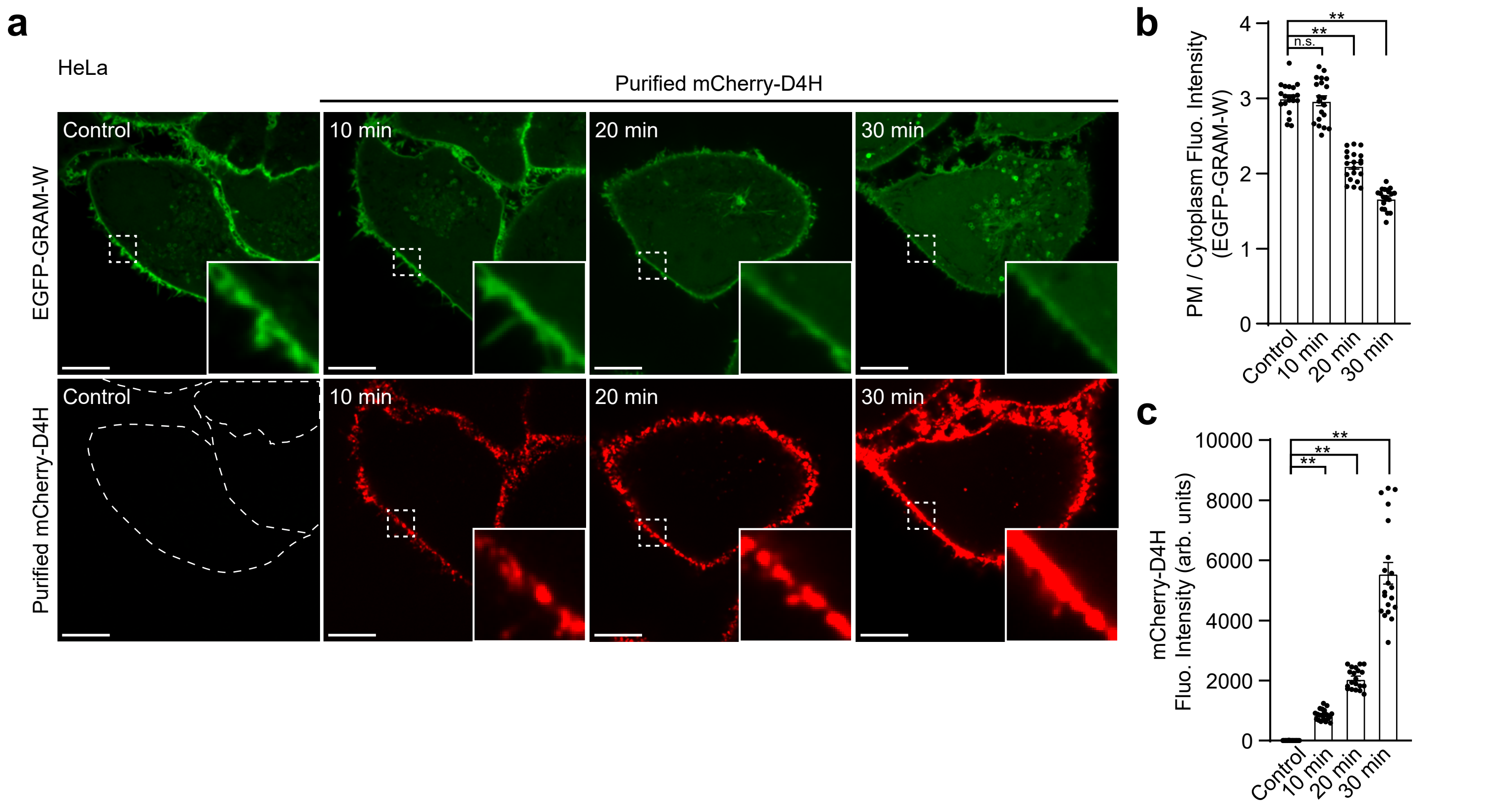

Supplementary Fig. 4. Dissociation of EGFP-GRAM-W from the PM induced by accessible cholesterol trapping via purified mCherry-D4H proteins. a. Confocal images of live HeLa cells stably expressing EGFP-GRAM-W that were stained with purified mCherry-D4H proteins (15 µg/ml) for indicated periods of time. White dotted lines are drawn to depict the PM. Insets show at higher magnification the regions indicated by white dashed boxes. Scale bars, 10 µm. **b.** Quantification of the ratio of PM signals to cytosolic signals of EGFP-GRAM-W, as assessed by confocal microscopy and line scan analysis as shown in (a) (mean ± SEM, n = 20 cells for each condition; data are pooled from two experiments; Dunnett's multiple comparisons test, **P < 0.0001. n.s. denotes not significant). **c.** Quantification of the mCherry-D4H signals, as assessed by confocal microscopy and line scan analysis as shown in (a) (mean ± SEM, n = 20 cells for each condition; data are pooled from two experiments; Dunnett's multiple comparisons test, **P < 0.0001).

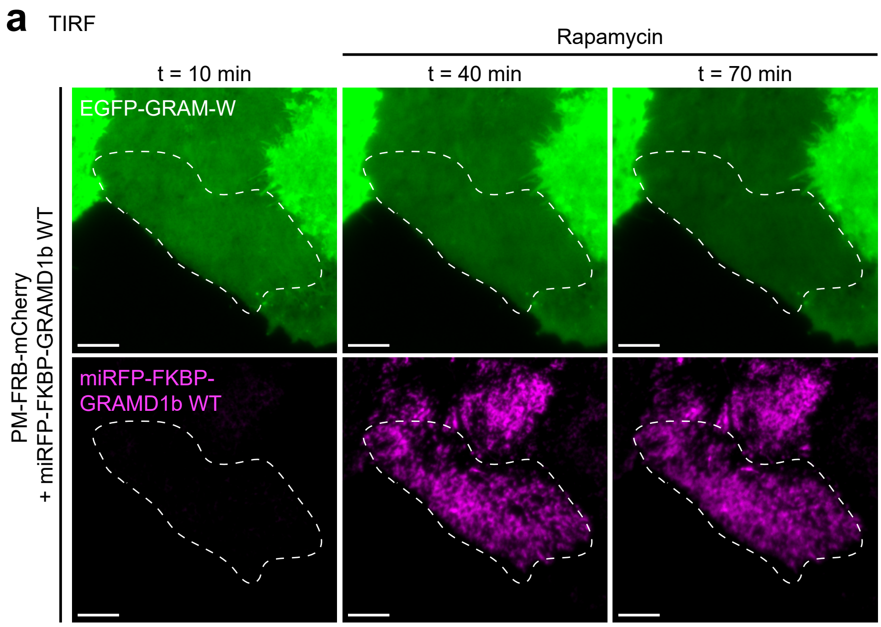

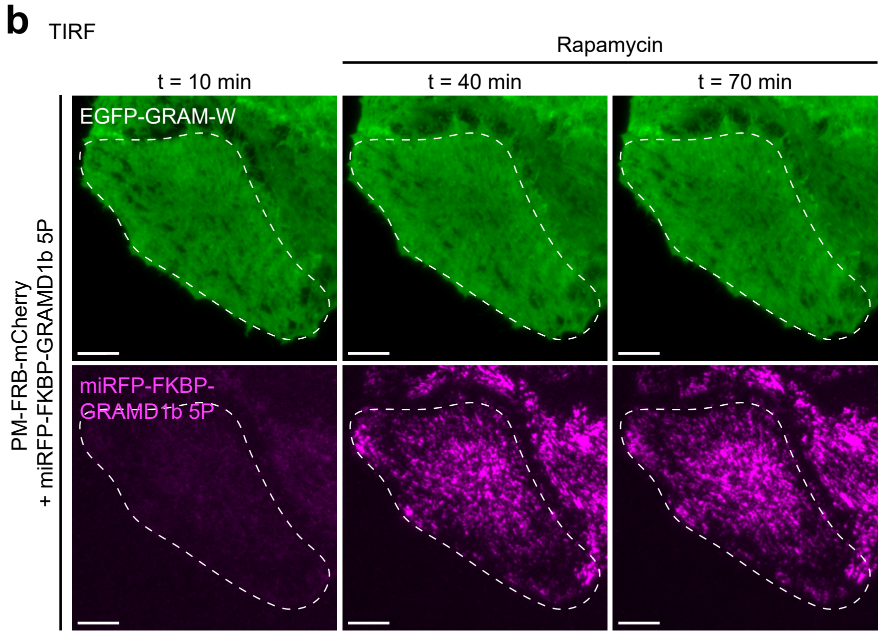

Supplementary Fig. 5. GRAM-W detects reduction of accessible PM cholesterol mediated by rapamycin-dependent recruitment of GRAMD1b to ER-PM contacts. a. TIRF images of live HeLa cells expressing EGFP-GRAM-W together with miRFP-FKBP-GRAMD1b (WT) and PM-FRB-mCherry. Cells were treated with rapamycin (200 nM) as indicated. White dotted lines are drawn to depict the PM. Note the gradual decrease in the signals of EGFP-GRAM-W at the PM upon rapamycin-induced recruitment of miRFP-FKBP-GRAMD1b (WT) to the PM. Quantification of normalized EGFP signals and that of normalized miRFP signals are shown in Fig. 3c, e, respectively. Scale bars, 10 μm. **b.** TIRF images of live HeLa cells expressing EGFP-GRAM-W together with miRFP-FKBP-GRAMD1b (5P) and PM-FRB-mCherry. Cells were treated with rapamycin (200 nM) as indicated. White dotted lines are drawn to depict the PM. Note the absence of significant changes in the signals of EGFP-GRAM-W at the PM upon rapamycin-induced recruitment of miRFP-FKBP-GRAMD1b (5P) to the PM. Quantification of normalized EGFP signals and that of normalized miRFP signals are shown in Fig. 3c, e, respectively. Scale bars, 10 μm.

a

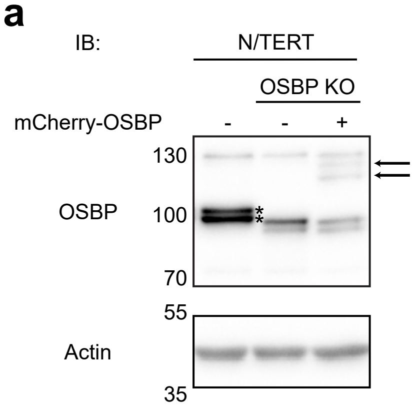

Supplementary Fig. 6. GRAM-W uncovers contribution of OSBP to accessible cholesterol distribution in keratinocytes. a. Lysates of wild-type N/TERT (Control), OSBP KO N/TERT, and OSBP KO N/TERT stably expressing mCherry-OSBP as indicated that were processed by SDS-PAGE and immunoblotted (IB) with anti-OSBP and anti-actin antibodies. Asterisks indicate endogenous OSBP; arrowheads indicate mCherry-OSBP. Experiments were repeated two times with similar results.

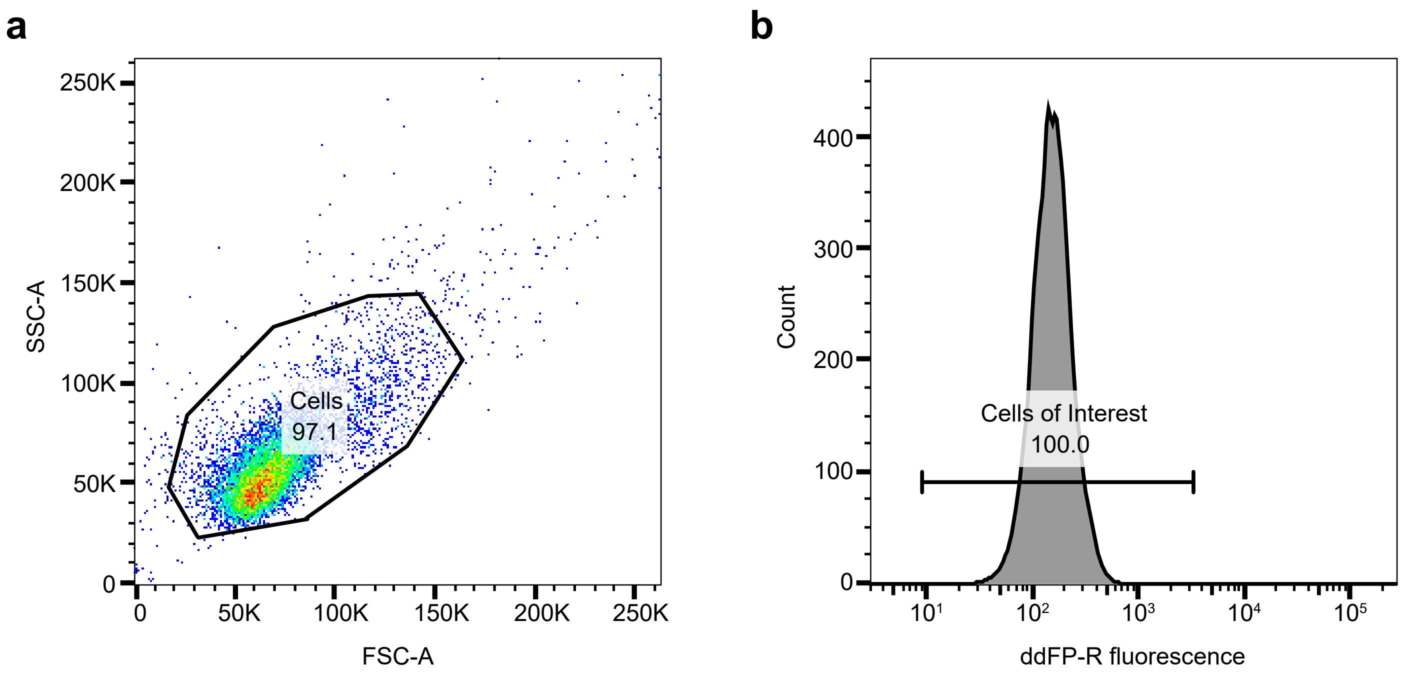

Supplementary Fig. 7. Gating strategy. a. The population of cells was gated in a forward vs side scatter plot. **b.** Cells from (a) were then displayed as a histogram showing the fluorescent intensity of ddFP-R-GRAM-W for Fig. 7i-k or ddFP-R-GRAM-H for Fig. 7l-n, following cell count normalization.

Supplementary Table 1

REAGENT or RESOURCE	SOURCE	IDENTIFIER
**Antibodies**		
Anti-OSBP (1:1,000 dilution for WB)	Sigma-Aldrich/Merck	RRID:AB_2676401
Anti-Actin (1:1,000 dilution for WB)	EMD Millipore	RRID:AB_2223041
Anti-SREBP-2 (1:1,000 dilution for WB)	Santa Cruz Biotechnology	RRID: AB_2194250
Anti-TH (1:500 dilution for IF)	Sigma-Aldrich/Merck	RRID: AB_297840
Anti-MAP2 (1:500 dilution for IF)	Sigma-Aldrich/Merck	RRID: AB_2138153
Goat anti-Rabbit IgG (H+L) Highly Cross-Adsorbed Secondary Antibody, Alexa Fluor™ 594 (1:500 dilution for IF)	Thermo Fisher Scientific	RRID: AB_2534095
Goat anti-Chicken IgY (H+L) Cross-Adsorbed Secondary Antibody, Alexa Fluor™ 633 (1:500 dilution for IF)	Thermo Fisher Scientific	RRID: AB_2535756
Goat Anti-Rabbit IgG (H+L)-HRP Conjugate (1:5,000 dilution for WB)	Bio Rad	RRID:AB_11125142
Goat Anti-Rabbit IgG (H+L)-HRP Conjugate (1:5,000 dilution for WB)	Bio Rad	RRID:AB_11125547
**Bacterial Strains**		
E. coli BL21-DE3 Rosetta		
**Chemicals, Peptides, and Recombinant Proteins**		
1,2-dioleoyl-sn-glycero-3-phosphocholine (DOPC)	Avanti Polar Lipids	850375
1,2-dioleoyl-sn-glycero-3-phospho-L-serine (sodium salt) (DOPS)	Avanti Polar Lipids	840035
2-Mercaptoethanol	Gibco	21985023
Ascorbic Acid	Stemcell Technologies	72132
B-27™ Supplement minus vitamin A	Gibco	12587010
Benzonase® Nuclease	Santa Cruz Biotechonology	sc-202391
Blasticidine S Hydrochloride	Sigma-Aldrich/Merck	15205
Bovine Pituitary Extract (BPE)	Gibco	17005042
CHIR99021	Tocris	4423/10
Cholesterol	Sigma-Aldrich/Merck	C8667
Colloidal Blue Staining Kit	Thermo Fisher Scientific	LC6025
cOmplete™, EDTA-free Protease Inhibitor Cocktail	Sigma-Aldrich/Merck	11873580001
Cultrex Stem Cell Qualified Human Fibronectin	RnD Systems	3420-001-03
Cultrex Stem Cell Qualified Laminin I	RnD Systems	3400-010-03
DAPT	Tocris	2634/10
Dibutyryl cAMP	Santa Cruz	sc-201567B
DMEM (4.5g/l Glucose) with L-Gln, without Sodium Pyruvate	Nacalai Tesque, Japan	08459-35
DNase	Sigma-Aldrich/Merck	DN25
Fetal Bovine Serum	Gibco	10270-106
Geneticin (G418 Sulfate)	Gibco	10131027
GlutaMAX™ Supplement	Gibco	35050061
HCS LipidTOX™ Red Neutral Lipid Stain, for cellular imaging	Thermofisher	H34476

HisPur™ Ni-NTA Resin	Thermofisher	88222
Human Recombinant EGF	Gibco	17005042
Isopropyl-1-thio-B-d-galactopyranoside (IPTG)	ThermoFisher	R0392
Keratinocyte SFM (1X)	Gibco	17005042
KnockOut™ DMEM/F-12	Gibco	12660012
KnockOut™ Serum Replacement	Gibco	10828010
LDN193189	Tocris	6053/10
Lipofectamine 2000 Reagent	Thermo Fisher Scientific	11668-019
Lipoprotein Deficient Serum (LPDS)	Sigma-Aldrich/Merck	S5394
Low Density Lipoprotein (LDL)	Stemcell technologies	02698
Lysozyme from chicken egg white	Sigma-Aldrich/Merck	62970
Matrigel™ hESC-Qualified Matrix, LDEV-free	Corning	354277
MEM Non-Essential Amino Acids Solution (100X)	Gibco	11140050
Methyl-β-cyclodextrin	Sigma-Aldrich/Merck	C4555
Mevastatin	Santa Cruz	sc-200853A
N-2 Supplement	Gibco	17502001
Neurobasal™ Medium	Gibco	21103049
Opti-MEM™ I Reduced Serum Medium	Thermo Fisher Scientific	31985070
OSW-1	Cayman Chemical	30310
Phorbol-12-myristate-13-acetate (PMA)	Sigma-Aldrich/Merck	P8139
Poly-L-Ornithine	Sigma-Aldrich	P4957
Purmorphamine	Tocris	4551/10
Puromycin	Stemcell	73342
Rapamycin	Sigma-Aldrich/Merck	R0395
Recombinant Human GDNF	Peprotech	450-10
Recombinant Human/Murine/Rat BDNF	Peprotech	450-02
Recombinant mouse sonic hedgehog/Shh (C25II)	RnD Systems	464-SH-025/CF
ReLeSR™	Stemcell Technologies	05872
RPMI 1640 with L-Gln and HEPES	Nacalai Tesque, Japan	30263-95
Sandoz 58-035	Sigma-Aldrich/Merck	S9318
SB431542	Tocris	1614/10
Sodium Bicarbonate (7.5%)	Gibco	25080094
Sphingomyelinase from Bacillus cereus (SMase)	Sigma-Aldrich/Merck	S9396
StemPro™ Accutase™	Gibco	A1110501
Streptomycin/Penicillin	Gibco	15140122
TeSR™-E8™	Stemcell Technologies	05990
TGF-β3	Peprotech	100-36E
Tris (2-carboxyethyl) phosphine (TCEP)	Sigma-Aldrich/Merck	C4706
U18666A	Sigma-Aldrich/Merck	U3633
Y-27632	Stemcell Technologies	72308

**Critical Commercial Assays**		
BCA Protein Assay Kit	NEB	23225
Lenti-X GoStix Plus	Takara Bio	631281
NEBuilder HiFi DNA Assembly Cloning Kit	NEB	E5520S
P3 Primary Cell 4D-Nucleofector™ X Kit S	Lonza	V4XP-3032
Q5 Site-Directed Mutagenesis Kit	NEB	E0552S
MycoGuard™ Mycoplasma PCR Detection Kit	GeneCopoeia, Inc.	MP004
**Experimental Models: Cell Lines**		
HeLa M	Others	Gift from Pietro De Camilli
HeLa M stably expressing EGFP-GRAM$_{1b}$ WT	This paper	
HeLa M stably expressing EGFP-GRAM-W	This paper	
HeLa M stably expressing ddFP-R-GRAM-H	This paper	
HeLa M stably expressing ddFP-R-GRAM-W	This paper	
COS-7	Others	Gift from Pietro De Camilli
HEK293T	Others	Gift from Nguan Soon Tan
U2OS	Others	Gift from Wenting Zhao
N/TERT	Others	Gift from James G. Rheinwald
N/TERT stably expressing EGFP-GRAM-W	This paper	
THP-1	Others	Gift from Franklin Lei Zhong
THP-1 stably expressing EGFP-GRAM-W	This paper	
asF5	CellResearch Corporation Pte Ltd	
**Oligonucleotides**		
CACTTCGTTTTGGGCCCGGGATA	This paper	GRAMD1b_G187W_F
AAGAAGTGCTTTTCTGAATCAGTGC	This paper	GRAMD1b_G187W_R
CAAACTATAGTCTCAATTTATGG	This paper	OSBP_S416S_F
GCTTGTATGGTATTCTGG	This paper	OSBP_S416S_R
TAATGAGCCCCTATCCATGCTTC	This paper	OSBP_L444L_F
AAGTTTACCGGCATGGGG	This paper	OSBP_L444L_R
CCGAGCTACCGGTCATGGTGAGCAAGAGCGAGGAGGTC	This paper	AgeI-RA-S
TTGAGCTCGAGAGGTACCCTTGTACAGCTCGTCCATG	This paper	XhoI-RA-AS

GGGCCTGAATTCGTGAGCAAGGGCGAGGAGACCATCAAAGAGTTCATGCGCTTCAAGGTGCGCATGGAGGGCTCCATGAACGGCCACGAGTTCGAGATCGAGGGCGAGGGCGAGGGCCGCCCCTACGAGGGCACCCAGACCGCCAAGCTGAAGGTGACCAAGGGCGGCCCCCTGCCCTTCGCCTGGGACATCCTGTCCCCCCAGTTCATGTACGGCTCCGAGGCGTACGTGAGGCACCCCGCCGACATCCCCGATTACAAGAAGCTGCCCTTCCCCGAGGGCTTCAAGTGGGAGCGCGTGATGAACTTCGAAGACGGCGGTCTGGTGACCGTTACCCAGGACTCCTCCCTGCAGGACGGCACGCTGATCTGCAAGGTGAAGATGCGCGGCACCAACTTCCCCCCCGACGGCCCCGTAATGCAGAAGAAGACCATGGGCTGGGAGGCCTCCACCGAGATGCTGTACCCCGAAGACGGCGTGCTGAAGGGCCATAGCTATCAGGCCCTGAAGCTGAAGGACGGCGGCCACTACCTGGTGGAGTTCGAGACCATCTACATGGCCAAGAAGCCCGTGCAACTGCCCGGCGATTACTGTGTGGACACCAAGCTGGACATCACCTCCCACAACGAGGACTACACCATCGTGGAACAGTACGGGCGCTCCGAGGGCCGCCACCGTCTGGGCATGGACGAGCGGTACAAGGGTACCAAGCAGAGAAATGAAGACTTCAGAAAGCTCTTTAAGCAGCTTCCAGACACGGAGCGCCTCATTGTTGATTACTCATGTGCACTCCAAAGAGACATTCTCCTTCAGGGCCGACTCTACCTCTCTGAAAATTGGATCTGCTTCTACAGCAACATCTTCCGCTGGGAAACTCTGCTGACAGTCCGTTTGAAAGACATCTGTTCCATGACTAAAGAAAAACAGCTCGCCTCATTCCCAATGCCATCCAAGTTTGCACTGATTCAGAAAAGCACTTCTTCACTTCGTTTCTCGCCCGGGATAGGACATATATGATGATGTTCCGGCTCTGGCAGAATGCTCTCCTTGAAAAGTGAGGATCCACCGGA	This paper	EcoRI_GB-GRAM-H_BamHI

GGGCCTGAATTCGTGAGCAAGGGCGAGGAGACCATCAAAGAGTTCATGCGCTTCAAGGTGCGCATGGAGGGCTCCATGAACGGCCACGAGTTCGAGATCGAGGGCGAGGGCGAGGGCCGCCCCTACGAGGGCACCCAGACCGCCAAGCTGAAGGTGACCAAGGGCGGCCCCCTGCCCTTCGCCTGGGACATCCTGTCCCCCCAGTTCATGTACGGCTCCGAGGCGTACGTGAGGCACCCCGCCGACATCCCCGATTACAAGAAGCTGCCCTTCCCCGAGGGCTTCAAGTGGGAGCGCGTGATGAACTTCGAAGACGGCGGTCTGGTGACCGTTACCCAGGACTCCTCCCTGCAGGACGGCACGCTGATCTGCAAGGTGAAGATGCGCGGCACCAACTTCCCCCCCGACGGCCCCGTAATGCAGAAGAAGACCATGGGCTGGGAGGCCTCCACCGAGATGCTGTACCCCGAAGACGGCGTGCTGAAGGGCCATAGCTATCAGGCCCTGAAGCTGAAGGACGGCGGCCACTACCTGGTGGAGTTCGAGACCATCTACATGGCCAAGAAGCCCGTGCAACTGCCCGGCGATTACTGTGTGGACACCAAGCTGGACATCACCTCCCACAACGAGGACTACACCATCGTGGAACAGTACGGGCGCTCCGAGGGCCGCCACCGTCTGGGCATGGACGAGCGGTACAAGGGTACCAAGCAGAGAAATGAAGACTTCAGAAAGCTCTTTAAGCAGCTTCCAGACACGGAGCGCCTCATTGTTGATTACTCATGTGCACTCCAAAGAGACATTCTCCTTCAGGGCCGACTCTACCTCTCTGAAAATTGGATCTGCTTCTACAGCAACATCTTCCGCTGGGAAACTCTGCTGACAGTCCGTTTGAAAGACATCTGTTCCATGACTAAAGAAAAACAGCTCGCCTCATTCCCAATGCCATCCAAGTTTGCACTGATTCAGAAAAGCACTTCTTCACTTCGTTTTGGGCCCGGGATAGGACATATATGATGATGTTCCGGCTCTGGCAGAATGCTCTCCTTGAAAAGTGAGGATCCACCGGA	This paper	EcoRI_GB-GRAM-W_BamHI
GCGCTACCGGTCGCCACCATGGTGAGCAAGGGCGAGGAGACC	This paper	AgeI-GB3-NS
CTGCAGAATTCTCACTTTTCAAGGAGAGCATTCTGCCAG	This paper	EcoRI-stop-GRAM1b-AS
CAGGGGGATCCACTTACCATGGTTCCTTTGTCTCAAGAAGAATCC	This paper	BamHI-BlastR-NS
TTAAAGGTACCTTAGCCCTCCCACACATAACCAGAG	This paper	KpnI-BlastR-CAS
AGGGGGATCCACTTACCATGATTGAACAAGATGGATTGCACGCAG	This paper	BamHI-NeoR-NS
TTAAAGGTACCTCAGAAGAACTCGTCAAGAAGGCGATAG	This paper	KpnI-NeoR-CAS
mA*mA*mU*rGrArCrUrUrGrArUrArGrCrUrArArGrCrArGrUrUrUrUrArGrArGrCrUrArGrArArArUrArGrCrArArGrUrUrArArArArUrArArGrGrCrUrArGrUrCrCrGrUrUrArUrCrArArCrUrUrGrArArArArArGrUrGrGrCrArCrCrGrArGrUrCrGrGrUrGrCmU*mU*mU*rU	This paper	OSBP-sgRNA#1
mG*mC*mU*rCrGrArGrGrGrUrUrUrCrUrUrCrCrArGrUrGrUrUrUrUrArGrArGrCrUrArGrArArArUrArGrCrArArGrUrUrArArArArUrArArGrGrCrUrArGrUrCrCrGrUrUrArUrCrArArCrUrUrGrArArArArArGrUrGrGrCrArCrCrGrArGrUrCrGrGrUrGrCmU*mU*mU*rU	This paper	OSBP-sgRNA#2
**Recombinant DNA**		
pNIC28-Bsa4 His-GRAM1b$_{92-207}$ (GRAM$_{1b}$)	Ercan et al (2021)	BE36
pNIC28-Bsa4 His-GRAM$_{1b}$ (G187L)	Ercan et al (2021)	BE99
pNIC28-Bsa4 His-GRAM$_{1b}$ (G187W)	This paper	D124
pNIC28-Bsa4 mCherry-D4H	Ercan et al (2021)	D148
EGFP-GRAM$_{1b}$	Naito et al (2019)	D2
EGFP-GRAM$_{1b}$ (G187L) (EGFP-GRAM-H)	Ercan et al (2021)	BE84

EGFP-GRAM$_{1b}$ (G187K)	Ercan et al (2021)	D59
EGFP-GRAM$_{1b}$ (G187W) (EGFP-GRAM-W)	Ercan et al (2021)	D60
EGFP-GRAM$_{1b}$ (G187R)	Ercan et al (2021)	D76
EGFP-GRAM$_{1b}$ (G187H)	Ercan et al (2021)	D77
EGFP-GRAM$_{1b}$ (G187D)	Ercan et al (2021)	D78
EGFP-GRAM$_{1b}$ (G187E)	Ercan et al (2021)	D79
EGFP-GRAM$_{1b}$ (G187S)	Ercan et al (2021)	D80
EGFP-GRAM$_{1b}$ (G187T)	Ercan et al (2021)	D81
EGFP-GRAM$_{1b}$ (G187N)	Ercan et al (2021)	D82
EGFP-GRAM$_{1b}$ (G187Q)	Ercan et al (2021)	D83
EGFP-GRAM$_{1b}$ (G187C)	Ercan et al (2021)	D84
EGFP-GRAM$_{1b}$ (G187P)	Ercan et al (2021)	D85
EGFP-GRAM$_{1b}$ (G187A)	Ercan et al (2021)	D86
EGFP-GRAM$_{1b}$ (G187V)	Ercan et al (2021)	D87
EGFP-GRAM$_{1b}$ (G187I)	Ercan et al (2021)	D88
EGFP-GRAM$_{1b}$ (G187M)	Ercan et al (2021)	D89
EGFP-GRAM$_{1b}$ (G187F)	Ercan et al (2021)	D90
EGFP-GRAM$_{1b}$ (G187Y)	Ercan et al (2021)	D91
PM-FRB-mCherry	Naito et al (2019)	TN133
miRFP-FKBP-GRAMD1b	Naito et al (2019)	TN60
miRFP-FKBP-GRAMD1b (5P)	Naito et al (2019)	TN67
EGFP-D4H	Naito et al (2019)	LK59
pLJM1-EGFP	Addgene	19319
pLJM1-EGFP-GRAM$_{1b}$	Ercan et al (2021)	TN208
pLJM1-EGFP-GRAM$_{1b}$ (G187L) (pLJM1-EGFP-GRAM-H)	Ercan et al (2021)	TN209
pLJM1-EGFP-GRAM$_{1b}$ (G187W) (pLJM1-EGFP-GRAM-W)	This paper	D150
mRuby-OSBP	Naito et al (2019)	D42
mCherry-C1	De Camilli lab	YS300
mCherry-OSBP	This paper	D184
mCherry-OSBP (CRISPR-R)	This paper	D204
pLJM1-mCherry-OSBP	This paper	D208
pEGFP-C1	De Camilli lab	YS276
RA-NES	Addgene	61019
Lck-mScarlet-I	Addgene	98821
pLJC6-3HXA-TMEM192	Addgene	104434
pLJM1-EGFP-BlastR	This paper	MY5
PM-RA	This paper	TN397
pLJM1-PM-RA-BlastR	This paper	MY11
pLJM1-EGFP-NeoR	This paper	MY6
B-GRAM-W	This paper	TN409
B-GRAM-H	This paper	TN408
pLJM1-B-GRAM-W-NeoR	This paper	MY10
pLJM1-B-GRAM-H-NeoR	This paper	MY9
pMD2.G	Addgene	12259
pRSV-REV	Addgene	12253
pMDL/pRRE	Addgene	12251
mCherry-C1	De Camilli lab	YS300
iRFP-P4M	De Camilli lab	YS313
mCherry-Lact-C2	De Camilli lab	YS182
iRFP-PH-PLCδ1	De Camilli lab	YS174
pLAMP1-miRFP703	Addgene	79998
LAMP1-mRFP-FLAG	Addgene	34611

**Software and Algorithms**		
Prism 8.0.1	Graph Pad	
PyMoL	The PyMOL Molecular Graphics System, Version 1.2r3pre, Schrödinger, LLC.	
ImageJ Fiji	Schneider et al., (2012)	https://imagej.nih.gov/ij/
**Other**		
100 nm NanoSizer Extruder	T&T Scientific	TT-002-0001
