# Supplementary material for: Visualization of accessible cholesterol using a GRAM domain-based biosensor
Source: Nat Commun. 2023 Oct 25;14:6773. doi: 10.1038/s41467-023-42498-7 (PMC10600248; doi:10.1038/s41467-023-42498-7)
Supplement: Supplementary file 3 — Description of Additional Supplementary Files [file 41467_2023_42498_MOESM3_ESM.pdf]

Description of Additional Supplementary Files

Supplementary Video Legends

Supplementary Video 1. GRAM-W detects LDL-derived accessible cholesterol in lysosomal membranes and the PM Live HeLa cells stably expressing either wild-type (WT) EGFP-GRAM1b (left) or EGFP-GRAMW (right) that were stimulated by low-density lipoprotein (LDL) (final concentration: 50 µg/ml) over a time course of 12 hrs were imaged under SDC microscopy. Images were taken every 15 mins upon addition of LDL. Image size, 55.0 µm x 55.0 µm.

Supplementary Video 2. GRAM-W detects reduction of accessible PM cholesterol mediated by rapamycin-dependent recruitment of GRAMD1b to ER-PM contacts Live HeLa cells expressing EGFP-GRAM-W (left; green) together with miRFP-FKBPGRAMD1b (WT) (right; magenta) and PM-FRB-mCherry, were imaged under TIRF microscopy. Images were taken every 1 min, and rapamycin (final concentration: 200 nM) was added at 10 min time point. Image size, 63.9 µm x 63.9 µm.

Supplementary Video 3. GRAM-W detects reduction of accessible PM cholesterol mediated by rapamycin-dependent recruitment of GRAMD1b to ER-PM contacts Live HeLa cells expressing EGFP-GRAM-W (left; green) together with miRFP-FKBPGRAMD1b (5P) (right; magenta) and PM-FRB-mCherry, were imaged under TIRF microscopy. Images were taken every 1 min, and rapamycin (final concentration: 200 nM) was added at 10 min time point. Image size, 63.9 µm x 63.9 µm.

Supplementary Video 4. Ultrasensitive detection of accessible PM cholesterol via ddFP-R-GRAM-W system Live HeLa cells expressing PM-RA and B-GRAM-W (i.e., ddFP-R-GRAM-W system) were imaged under SDC microscopy. Images were taken every 1 min, and MCD (final concentration: 5 mM) was added at 5 min time point. Image size, 55.0 µm x 55.0 µm.

Supplementary Video 5. Ultrasensitive detection of accessible PM cholesterol via ddFP-R-GRAM-H system Live HeLa cells expressing PM-RA and B-GRAM-H (i.e., ddFP-R-GRAM-H system) were imaged under SDC microscopy. Images were taken every 1 min, and MCD/Chol (final concentration: 200 µM) was added at 5 min time point. Image size, 55.0 µm x 55.0 µm.
